# Antenatal Corticosteroids for Late-Preterm Infants: A Decision-Analytic and Economic Analysis

**DOI:** 10.5402/2012/491595

**Published:** 2012-12-27

**Authors:** Jamie A. Bastek, Holly Langmuir, Laxmi A. Kondapalli, Emmanuelle Paré, Joanna E. Adamczak, Sindhu K. Srinivas

**Affiliations:** ^1^Maternal and Child Health Research Program, Department of Obstetrics & Gynecology, Center for Research on Reproduction and Women's Health, Perelman School of Medicine, University of Pennsylvania, Philadelphia, PA 19104, USA; ^2^Division of Maternal-Fetal Medicine, Department of Obstetrics and Gynecology, Hospital of the University of Pennsylvania, 585 Dulles Building, 3400 Spruce Street, Philadelphia, PA 19104, USA; ^3^Section of Reproductive Endocrinology and Infertility, Department of Obstetrics & Gynecology, University of Colorado, Anschutz Medical Campus, Denver, Aurora, CO 80013, USA; ^4^Division of Maternal-Fetal Medicine, Department of Obstetrics & Gynecology, University of British Columbia, Vancouver, BC, Canada V6T 1Z4; ^5^Division of Maternal-Fetal Medicine, Department of Obstetrics & Gynecology, Rady Children's Hospital, San Diego, CA 92123, USA

## Abstract

*Objectives*. Antenatal corticosteroids (ACS) are not routinely administered to patients at risk for delivery between 34 and 36 6/7 weeks. Our objective was to determine whether ACS are cost-effective for late-preterm infants at risk for imminent preterm delivery. We hypothesized that the preferred strategy <36 weeks would include ACS while the preferred strategy ≥36 weeks would not. *Methods*. We performed decision-analytic and cost-effectiveness analyses to determine whether ACS was cost-effective at 34, 35, and 36 weeks. We conducted a literature review to determine probability, utility, and cost estimates absent of patient-level data. Base-case cost-effectiveness analysis, univariable sensitivity analysis, and Monte Carlo simulation were performed. A threshold of $100,000/QALY was considered cost-effective. *Results*. The incremental cost-effectiveness ratio favored the administration of a full course of ACS at 34, 35, and 36 weeks ($62,888.25/QALY, $64,425.67/QALY, and $64,793.71/QALY, resp.). A partial course of ACS was not cost-effective. While ACS was the consistently dominant strategy for acute respiratory outcomes, all models were sensitive to changes in variables associated with chronic respiratory disease. *Conclusions*. Our findings suggest that the administration of ACS to patients at risk of imminent delivery 34-36 weeks could significantly reduce the cost and acute morbidity associated with late-preterm birth.

## 1. Introduction

Preterm birth (PTB) is a leading cause of neonatal morbidity and mortality, and it is well recognized that the risk decreases with increasing gestational age of delivery. The benefits of antenatal corticosteroid (ACS) administration to patients at risk of delivery <34 weeks to mitigate the risks of prematurity have been well researched [1, 2]. As a result, the American Congress of Obstetricians and Gynecologists (ACOG) recommends ACS to patients between 24 and 33 6/7 weeks at risk of PTB [2, 3]. They specifically, recommend either two 12 mg doses of betamethasone given 24 hours apart or four 6 mg doses of dexamethasone given 12 hours apart to reduce the incidence of respiratory distress syndrome, intraventricular hemorrhage, necrotizing enterocolitis, and/or neonatal death [2, 3].

Late-preterm infants, delivered 34–36 6/7 weeks, are at less risk of adverse outcomes compared to infants delivered at earlier gestational ages. Despite earlier beliefs that outcomes for these infants were not much different from term infants [4] and that fetal lungs reach surfactant maturity by 34 weeks [5], recent evidence has revealed that late-preterm infants face significantly *greater* risk of adverse respiratory outcomes compared to term infants [6–9]. Nevertheless, there are minimal data to suggest whether ACS might benefit patients who need delivery at 34–36 6/7 weeks [10]. As a result, current ACOG guidelines do not recommend ACS at these gestational ages.

Given the prevalence of acute respiratory morbidity between 34 and 36 6/7 weeks and the biologic plausibility for steroids to be beneficial at these gestational ages, our objective was to use decision-analytic and cost-effectiveness analysis to determine whether ACS is cost-effective in late-preterm infants at risk of delivery. Due to the decreasing prevalence of acute respiratory morbidity with increasing gestational age, we hypothesized that the preferred strategy for infants <36 weeks would include ACS while the preferred strategy for infants ≥36 weeks would not.

## 2. Methods

### 2.1. Decision Tree Model

Decision-analytic and cost-effectiveness analysis is designed to assess the costs and utilities of alternative strategies compared to a model of usual practice. Although absent of patient-level data, this form of analysis integrates existing data from the literature in an effort to make population-level healthcare decisions [11].

**Figure 1:**
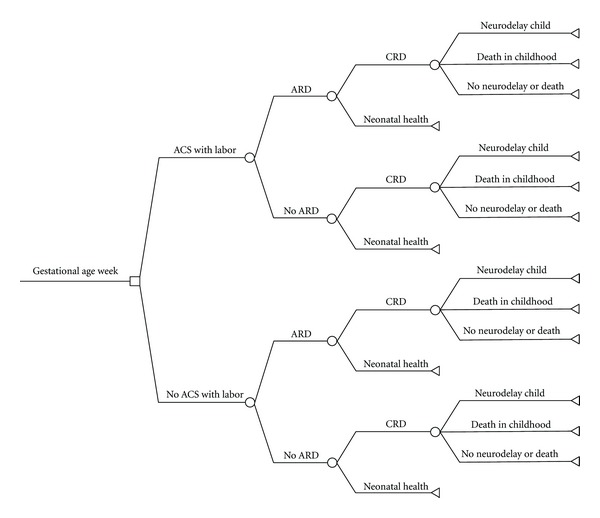
Decision tree demonstrating the relationship between possible treatment strategies and outcomes. ACS: antenatal corticosteroids; ARD: acute respiratory disease; CRD: chronic respiratory disease.

Prior to performing our analyses we constructed a decision tree—a visual tool to illustrate the relationship between possible treatment strategies and outcomes (Figure 1).

Three separate decision trees were created, one to represent each gestational age week during the late-preterm period (34, 35, and 36 weeks). Models were constructed for women with singleton pregnancies who present and subsequently deliver within the same gestational age week. The choice node of each tree involved the decision of whether or not to administer ACS to patients at risk of PTB, as the latter represents the currently accepted standard of care at these gestational ages.

In either strategy, we assumed that the infant might or might not experience acute and/or chronic respiratory disease. Furthermore, we assumed that the infant might also experience nonrespiratory outcomes including adverse neurodevelopment, death, or health. We chose these outcomes because we believed that these were the most medically relevant outcomes to late-preterm infants and because there are data in the literature to support that ACS could mitigate their occurrence.

Adverse maternal outcomes that might occur secondary to ACS—including fever requiring antibiotics, admission to the intensive care unit, hypertension, and/or side-effects of treatment—were not included in the models because they were thought to be very rare events and not likely due to the administration of ACS alone [2]. Furthermore, other potential adverse neonatal outcomes that may occur with ACS—including decreased weight, height, head circumference, hypothalamic-pituitary-adrenal axis function, educational attainment, and/or intellectual parameters, increased blood pressure and/or cholesterol, or visual and/or hearing impairments—were not included based upon their insignificant association with ACS [2].

### 2.2. Data Sources: Probabilities and Relative Risks

We conducted a literature review to estimate the probabilities of relevant neonatal outcomes, namely, acute and/or chronic respiratory disease, adverse neurodevelopment, death, or health, both with and without ACS (Tables 1 and 2).

We defined acute respiratory disease as a diagnosis of respiratory distress syndrome (RDS) and estimated the baseline prevalence of RDS among infants delivered at 34 weeks [2, 6, 12]. Probabilities and relative risks (RR) of acute respiratory disease among infants *not exposed* to corticosteroids were estimated using data from a multicenter observational study of more than 20,000 neonates who were not routinely administered corticosteroids [13]. To find gestational age-specific relative risks of RDS among infants *exposed *to corticosteroids we used data from the 2010 Cochrane review on antenatal corticosteroids [2] which provided information on the risk of RDS in infants exposed to corticosteroids at various gestational ages compared to infants not exposed to corticosteroids. We specifically, used data for “babies born ≥34 weeks” to represent 34-week deliveries, “babies born <36 weeks” to represent 35-week deliveries, and “babies born ≥36 weeks” to represent 36-week deliveries.

The baseline prevalence of chronic respiratory disease was estimated by using the prevalence of current asthma [14]. Since gestational age-specific data for chronic respiratory disease was not available, we used the relative risk of chronic respiratory disease associated with ACS exposure in “all babies” to estimate the risk at all gestational ages [2].

We then determined the baseline prevalence estimates as well as probabilities and relative risks of long-term, nonrespiratory childhood outcomes [15, 16]. Infant mortality rates were estimated using 2009 United States Center for Disease Control data (6.42 infant deaths/1000 live births) [15]. The rate of significant neurodevelopmental delay was estimated by using the prevalence of cerebral palsy from 1991–2000 New England data of singleton infants (2.46 cases of cerebral palsy/1000 neonatal survivors) [16]. Because gestational age-specific relative risks were not available for death or neurodevelopmental delay in childhood, we used the relative risk of each outcome associated with ACS exposure in “all babies” to estimate the risk at all gestational ages [2].

### 2.3. Data Sources: Utilities and Durations of Effect

We used information derived from the literature to determine the utility in quality-adjusted life years (QALYs) and duration of effect of the various neonatal outcomes (Table 3).

A health utility value, ranging from 0 (death) to 1 (perfect health), was assigned based upon published data quantifying perceived preferences for various health states [15, 17, 20, 21, 24]. All parameters were reviewed by an expert panel.

QALYs were computed by multiplying the duration of effect (anticipated number of life years in each health state) by the utility associated with that health state. We assumed that utilities were independent and weighed equally.

Utilities for neonatal outcomes were estimated based upon a study of over 4,000 parent and guardian preferences of various pediatric morbidities. Because the utility of a diagnosis of RDS has not specifically been previously studied, we decided that 10-day intensive care unit admission was an acceptable alternative since infants with RDS are managed in the neonatal intensive care unit. Applied utilities for both chronic respiratory disease and adverse neurodevelopment were those for moderate persistent asthma [17] and moderate cerebral palsy [17], respectively.

The duration of effect of acute childhood illness was estimated based upon latency to 40 weeks gestation (6, 5, and 4 weeks duration for 34, 35, and 36 week infants, resp.) [17]. The duration of effect of child health, chronic lung disease, and death in childhood were estimated to be 78.2 years based upon 2009 life expectancy data [15]. Duration of effect of neurodevelopmental delay in childhood was estimated to be 17 years based upon data describing the life expectancy for patients with cerebral palsy [16].

### 2.4. Data Sources: Cost

Finally, we estimated the costs in US dollars of various neonatal outcomes as well as the cost of potential medical interventions [18–20, 22, 23].

Of note, we estimated that the cost of ACS was more than simply the cost of the medication. Instead, we assumed that patients who would receive ACS—like most patients <34 weeks who currently receive ACS—would require inpatient management due to either their risk of relatively imminent spontaneous PTB or their need for medical attention prior to iatrogenic PTB. Inpatient ACS therapy is associated with approximately 48 hour of hospitalization *prior to delivery.* Thus ACS potentially doubles the total length of hospital stay compared to a patient without ACS who has an uncomplicated spontaneous vaginal delivery and is discharged on postpartum day two [22, 23].

All cost estimates were made from a single payer perspective and adjusted to 2011 US dollars. Future costs were discounted to using a 3% annual discount rate [25].

### 2.5. Analyses

When more than one data source was available to describe a health state, the point estimate was calculated as a weighted mean by sample size and the range was represented by the lowest and highest values from published sources. When estimates were derived from a single source, the range was defined by the 95% confidence interval that was calculated from the binomial distribution.

We performed base-case cost-effectiveness analysis to calculate incremental cost-effectiveness ratios (ICERs) to compare strategies within each gestational age week. Since many infants will deliver prior to the completion of 48 hours of steroid prophylaxis at these gestational ages, we next evaluated the cost effectiveness of steroid administration if a full course was not administered. We adjusted our estimates and repeated our base-case analyses to determine the preferred strategy associated with receiving only a partial course of steroids at each gestational age week of delivery. We assumed that one dose of steroids resulted in only a 50% reduction in the rate of complications and was associated with a 50% reduction in hospital costs. We then studied a hypothetical cohort of late-preterm, singleton infants to determine both the number of adverse events that could be prevented as well as the cost that could be saved with the administration of ACS to all patients at risk of delivery at a particular gestational age.

Univariable sensitivity analyses were performed to determine whether varying each estimate of probability, utility, and/or cost impacted which strategy was considered optimal.

Finally, we replaced point estimates for each probability variable with beta distributions (due to the dichotomous nature of all probabilities in the model) and point estimates for each cost and utility variable with normal distributions. Then, 10,000-iteration second-order Monte Carlo simulation (probabilistic sensitivity analysis) was performed to simultaneously and randomly vary all variables across their plausible ranges to determine over what range of values we could be 95% confident that one strategy was of good value compared to the alternative. These calculations were also used to determine how frequently the optimal strategy was consistent with the results of the base-case analysis.

For all analyses, we chose the standard and accepted threshold of $100,000/QALY as the willingness-to-pay threshold for all models [26]. Statistical analyses were performed using TreeAge Pro 2009 Suite software (TreeAge Software Inc. Williamstown, MA) and Stata version 10.1 (College Station, TX).

This study did not involve human subjects. Therefore, it was considered exempted by the Institutional Review Board at the Hospital of the University of Pennsylvania.

## 3. Results

At 34, 35, and 36 weeks of gestational age, the administration of ACS to patients at risk of imminent PTB was the more cost-effective strategy and was dominant compared to not administering ACS to those at risk of PTB (Table 4).

Given that many infants will be delivered prior to the completion of 48 hours of ACS prophylaxis, we then adjusted our estimates and repeated our base-case analyses to determine the preferred strategy associated with receiving only a partial course of ACS at each gestational age week of delivery. For late-preterm infants, the administration of a partial course of ACS with labor was not cost-effective at any gestational age week (ICER $131,233.39/QALY, $133,117.42/QALY, and $133,654.76/QALY at 34, 35, or 36 weeks, resp.).

Next, we studied a hypothetical cohort of late-preterm, singleton infants to determine both the number of acute (Table 5) and chronic (Table 6) adverse events that could be prevented as well as the cost that could be saved with administering ACS to all late-preterm patients at risk of delivery. Based on 2008 United States census data, the live birth rate is approximately 4.25 million infants per year of which 7.8% are late-preterm singletons [15]. Therefore, there are approximately 331,500 late-preterm singleton infants born annually in the United States. Under our base-case assumption, if each infant in this hypothetical cohort received ACS, the rate of RDS would decrease by approximately 50% for both 34 and 35 week infants (from 14,498 to 7,072 cases at 34 weeks and from 7,072 to 3,536 cases at 35 weeks) and by 40% (from 3,536 to 2,122 cases) for 36 week infants. While this would generate savings of approximately $32 million for 34 and 35 week infants, it would be associated with a loss of $3.4 million for 36-week infants due to the relatively greater cost of hospitalization associated with universal steroid therapy compared with the cost saved by reducing acute respiratory disease at this gestational age given its low prevalence (Table 5). Finally, ACS could also potentially save $166.2 million annually due to a reduction in chronic medical comorbidities (Table 6).

One-way sensitivity analyses were performed for all probabilities, costs, and utilities in each gestational age week tree. Despite variations in all variables associated with acute respiratory disease across their plausible ranges, steroid administration was the consistently preferred strategy. However, when sensitivity analyses were performed with variables associated with long-term adverse outcomes across their plausible ranges, all models were sensitive to changes in variables associated with chronic respiratory disease such that small changes in probabilities and utilities resulted in an efficacy of treatment that no longer justified the cost. At 34, 35, and 36 weeks, specifically if the probability of chronic respiratory disease with ACS was ≥6.45%, or the probability of chronic respiratory disease without ACS was ≤6.60%, or the utility associated with chronic respiratory disease was ≥94.0%, then the efficacy of treatment with ACS was no longer sufficient to justify the cost and the ICER exceeded the willingness-to-pay threshold.

Ten-thousand iteration second-order Monte Carlo simulation (probabilistic sensitivity analysis) was performed to simultaneously and randomly vary all acute and chronic disease distributions across their plausible ranges to determine for which values we could be 95% confident that one strategy was of good value compared to the alternative. At 34, 35, and 36 weeks, ACS was the dominant strategy—resulting in greater effectiveness for <$100,000/QALY—only 47.35%, 47.33%, and 46.98% of the time, respectively. Furthermore, at each gestational age week there was no willingness-to-pay threshold for which we could be 95% confident that the two therapies—ACS administration and nonadministration—differed significantly in value.

When analyses were restricted to only those distributions associated with acute respiratory disease, we found that in 100% of the 10,000 iterations, ACS was the dominant strategy resulting in greater effectiveness for <$100,000/QALY at 34, 35, and 36 weeks gestational age. Furthermore, we could be 95% confident that ACS represented good value compared to the alternative strategy of not administering ACS for all willingness-to-pay thresholds >$64,677, >$65,700, and >$65,819 at 34, 35, and 36 weeks, respectively.

## 4. Discussion

To date, we are the first group to approach the question of whether to administer ACS to late-preterm infants at risk of PTB through a decision-analytic and cost-effectiveness analysis. Our findings suggest that, contrary to our hypothesis regarding the cost-effectiveness of steroids at 36 weeks, under our base-case assumptions, a full course of ACS is cost-effective for patients 34–36 weeks at risk of PTB. Furthermore, our sensitivity analyses and Monte Carlo simulations confirm that, when the goal is to reduce *acute* respiratory disease, ACS is the dominant strategy. However, ACS to prevent *chronic* respiratory disease does not appear cost-effective.

A review of the literature reveals a single Brazilian randomized controlled trial from 2011 in which 320 women with late-preterm pregnancies at risk of imminent PTB were randomized to receive ACS or placebo [10]. Although powered to see a 50% difference between exposure groups, ACS did not reduce the incidence of respiratory disorders in late-preterm infants. However, the authors acknowledge that the results of their small, single-centered trial should not be interpreted in isolation. Instead, they recommend the incorporation of their findings into future meta-analyses and Cochrane reviews to draw more robust conclusions [10].

There are benefits to performing decision-analytic and cost-effectiveness analysis. This manner of data modeling allows for the generation of pretrial data without incurring the cost or time associated with a clinical study. Furthermore, through sensitivity analysis and Monte Carlo simulation, one can determine whether modifications in point estimates that might occur in various clinical scenarios and/or patient populations could alter the preferred strategy.

Our study was not without limitations. By nature of the decision-analytic model, assumptions regarding probability, utility, and cost of various disease states must be made. The strength of the decision-analytic model and the validity of the results are intrinsically associated with the accuracy of these assumptions. While the quality of the literature we reviewed reassured us regarding the accuracy of our assumptions, there were cases in which gestational age-specific information was not available. As a result, we had to assume that the point estimate referred to infants of all gestational ages. Furthermore, the data used to estimate the probabilities of acute respiratory disease among late-preterm infants exposed to ACS included data from steroid administration that did not occur immediately prior to delivery. However, if the mechanism of action of ACS in late-preterm infants is similar to that of preterm infants <34 weeks, this will bias our results towards the null.

In our model, all patients were assumed to deliver within the gestational age week in which they presented and all health states and disease pathways were seen as absolute, which we recognize may not reflect real-life disease processes.

Finally, although decision analysis yields results in a cost to benefit ratio, this value may not be what determines whether an intervention is deemed medically appropriate on an individual or population level.

While awaiting the results of the ongoing, large, multicenter, Maternal-Fetal Medicine Units Network trial (ALPS Trial-Antenatal Late Preterm: Randomized Placebo-Controlled Trial), our findings suggest that ACS to patients at risk of delivery between 34 and 36 weeks could significantly reduce both the cost and acute morbidity associated with late-preterm births.

## Figures and Tables

**Table 1:** Probabilities of acute adverse neonatal outcomes.

Adverse event	34 weeks	35 weeks	36 weeks
With corticosteroids			

Acute respiratory disease	0.064 [2, 6, 12] (0.02–0.104)	0.032 [2, 6, 12, 13] (0.01–0.052)	0.0192 [2, 6, 12, 13] (0.006–0.0312)

Without corticosteroids			

Acute respiratory disease	0.1312 [2, 6, 12, 13] (0.041–0.2132)	0.064 [2, 6, 12, 13] (0.02–0.104)	0.032 [2, 6, 12, 13] (0.01–0.052)

**Table 2:** Probabilities of long-term, adverse neonatal outcomes.

Adverse event	Baseline risk without steroids	Risk 34–36 weeks with steroids
Chronic respiratory disease	0.07 [14] (0.03–0.14)	0.0602 [2, 14] (0.0258–0.1204)
Death in childhood	0.00642 [15] (0.0059–0.0069)	0.0043656 [2, 15] (0.004012–0.004692)
Neurodevelopmental delay in childhood	0.00246 [16] (0.0022–0.0028)	0.0015744 [2, 16] (0.001408–0.001792)

**Table 3:** Utility and cost estimates of neonatal health states.

Variable	Point estimate (range)	Reference
Acute respiratory disease		
Utility	0.87 (0.79–0.93)	[17]
Cost ($)		
34 weeks	2,505 (334–50,657)	
35 weeks	1,081 (315–31,866)	[18]
36 weeks	863 (305–18,370)	

Chronic respiratory disease		
Utility	0.88 (0.80–0.94)	[17]
Cost ($)	56,641 (5,919–74,217)	[19]

Neurodevelopmental delay in childhood		
Utility	0.76 (0.66–0.84)	[17]
Cost ($)	270,790 (135,395–541,582)	[20]

Death in childhood		
Utility	0.01 (0.001–0.02)	[21]
Cost ($)	56,500 (27,960–83,881)	[22]

Child health		
Utility	1.00 (1.00)	[17]
Cost ($)	0.00 (0.00)	

Delivery cost		
Without ACS ($)	8,449 (5452–13,980)	[22]
With ACS ($)	16,277 (11,414–17,628)	[23]

ACS: antenatal corticosteroids.

**Table 4:** Base-case cost-effectiveness analysis comparison of ACS with labor to usual care.

GA weeks	Strategy	Cost ($)	IC ($)	Efficacy (Utiles)	IE (Utiles)	C/E ($/Utile)	ICER ($/QALY)	Interpretation
34	No ACS	12.8 K		155.811		82.24		Dominant
	ACS	19.9 K	7.1 K	155.923	0.112	127.55	62,888.25	

35	No ACS	12.6 K		155.792		80.59		Dominant
	ACS	19.8 K	7.2 K	155.904	0.112	126.76	64,425.67	

36	No ACS	12.5 K		155.774		80.33		Dominant
	ACS	19.7 K	7.2 K	155.885	0.112	126.66	64,793.71	

ACS: antenatal corticosteroids; GA: gestational age; IC: incremental cost; IE: incremental efficacy; QALY: quality adjusted life year.

**Table 5:** Number of cases of acute respiratory disease prevented and dollars saved through administration of ACS to hypothetical late-preterm birth cohort, assuming base-case estimates and late-preterm birth rate approximately 331,500 infants.

Strategy	Cases ARD (*n*)*	Cases ARD prevented (*n*)*	Cost saved ($ millions)
34 weeks ACS with labor	7072	7426	26.0
34 weeks No ACS (Reference)	14498	Reference	Reference
35 weeks ACS with labor	3536	3536	6.0
35 weeks No ACS (Reference)	7072	Reference	Reference
36 weeks ACS with labor	2122	1414	−3.4
36 weeks No ACS (Reference)	3536	Reference	Reference

*Assumes that prevalence of late-preterm live singletons delivered annually (≈331,500) is divided equally between each gestational age week.

ACS: antenatal corticosteroids; ARD: acute respiratory disease.

**Table 6:** Number of cases of chronic disease prevented and dollars saved through administration of ACS to hypothetical late-preterm birth cohort, assuming base-case estimates and late-preterm birth rate approximately 331,500 infants.

Outcomes	No ACS with labor (Reference)	ACS with labor
Chronic respiratory disease		

Cases (*n*)	23205	19956
Case prevented (*n*)	Reference	3249
Cost saved ($ millions)	Reference	55.3

Neurodevelopmental delay		

Cases (*n*)	816	522
Cases prevented (*n*)	Reference	294
Cost saved ($ millions)	Reference	78.0

Death in childhood		

Cases (*n*)	2128	1447
Cases prevented (*n*)	Reference	681
Cost saved ($ millions)	Reference	32.9

ACS: antenatal corticosteroids.
